# Peer Review of “Development and Assessment of a Point-of-Care Application (Genomic Medicine Guidance) for Heritable Thoracic Aortic Disease”

**DOI:** 10.2196/63646

**Published:** 2024-10-08

**Authors:** Jolyn Hersch

**Affiliations:** 1 Faculty of Medicine & Health, School of Public Health, The University of Sydney, Camperdown, Australia

**Keywords:** genomic medicine, point of care, thoracic aortic aneurysm, aortic dissection, decision support

*This is a peer-review report for “Development and Assessment of a Point-of-Care Application (Genomic Medicine Guidance) for Heritable Thoracic Aortic Disease.”*

## Round 1 Review

### General Comments

This paper [1] describes an application, Genomic Medicine Guidance, a point-of-care tool to deliver concise clinical information about gene mutations that cause heritable cardiovascular diseases.

### Specific Comments

This paper provides technical details about the application and its purpose, which is to collate data about genetic/genomic risks into a readily usable summary of clinical recommendations, particularly for nonexpert clinicians. This seems like a useful resource that could be updated and expanded as time goes on. I have some comments for the authors to address in order to strengthen the manuscript.

#### Major Comments

1. Section 3.6: Please add a little more information about the user reviews. Who were these application users? Did they have genetics training/expertise? When and how were they recruited to provide this feedback? Was their feedback acted on in any way? (These details may belong better in the Methods section.)

2. Line 102: The text refers to Table 1, but I cannot find any table.

3. Line 115: Producing “patient-friendly outputs” is much easier said than done. How did the developers ensure that outputs are actually patient friendly? Were patients involved in the project team and/or user-testing activities? Are outputs “friendly” for patients from diverse sociodemographic populations or only those with high literacy and education? Are any visual/graphical formats used in addition to text? After jotting down the queries above, I explored the website a bit myself. I would not consider the “patient friendly results” to be very friendly at all, especially for those with lower health literacy. It would be good to spell out in the manuscript exactly what steps have been taken in this direction so far and to acknowledge that there is more that could—and hopefully will—be done.

4. Line 167: Related to the above comments, the manuscript concludes with a throwaway line that the application “empowers patients to take an informed role in healthcare decisions.” Undoubtedly, the application developers aspire to “empower” patients, but the manuscript presents no evidence to back this up. If the authors have data on how the app affects patient-reported outcome measures, please add this into the manuscript. If not, please temper this concluding statement. Perhaps an evaluation of whether and how the application actually changes patient knowledge and participation in decision-making would be a valuable next step in the research agenda.

#### Minor Comments

5. Line 42‐43: Please provide brief examples of “timely individualized interventions that can prevent deaths.”

6. Line 44: Define “ACC/AHA.

7. Line 105: Define “HTAD.”

## Round 2 Review

My comments were addressed to my satisfaction.
